# Screening of genes involved in interactions with intestinal epithelial cells in *Cronobacter sakazakii*

**DOI:** 10.1186/s13568-016-0246-4

**Published:** 2016-09-15

**Authors:** Xin-jun Du, Xia Zhang, Ping Li, Rui Xue, Shuo Wang

**Affiliations:** 1 Key Laboratory of Food Nutrition and Safety, Ministry of Education, Tianjin University of Science and Technology, Tianjin, 300457 China; 2 Tianjin Entry-Exit Inspection and Quarantine Bureau, Tianjin, 300461 China

**Keywords:** *Cronobacter sakazakii*, Epithelial cell, Adhesion, Invasion, Genes

## Abstract

*Cronobacter sakazakii* possesses a significant ability to adhere to and invade epithelial cells in its host. However, the molecular mechanisms underlying this process are poorly understood. In the current study, the adhesive and invasive capabilities of 56 *C. sakazakii* strains against human epithelial cells were evaluated, and one of them was selected for construction of a mutant library using the Tn5 transposon. In a systematic analysis of the adhesive and invasive capabilities of 1084 mutants, 10 mutants that showed more than a 50 % reduction in adhesion or invasion were obtained. Tail-PCR was used to sequence the flanking regions of the inserted transposon and 8 different genes (in 10 different mutants) were identified that encoded an exonuclease subunit, a sugar transporter, a transcriptional regulator, two flagellar biosynthesis proteins, and three hypothetical proteins. Raman spectroscopy was used to analyze variations in the biochemical components of the mutants, and the results showed that there were fewer amide III proteins, protein -CH deformations, nucleic acids and tyrosines and more phenylalanine, carotenes, and fatty acids in the mutants than in the wild type strain. Real-time PCR was used to further confirm the involvement of the genes in the adhesive and invasive abilities of *C. sakazakii*, and the results indicated that the expression levels of the 8 identified genes were upregulated 1.2- to 11.2-fold. The results of this study provide us with insight into the mechanism by which *C. sakazakii* infects host cells at molecular level.

**Electronic supplementary material:**

The online version of this article (doi:10.1186/s13568-016-0246-4) contains supplementary material, which is available to authorized users.

## Introduction

*Cronobacter* is a genus of Gram-negative, rod-shaped, non-spore-forming bacteria that belong to the family Enterobacteriaceae. It is widely accepted that this genus contains seven species, including *Cronobacter sakazakii*, *Cronobacter malonaticus*, *Cronobacter turicensis*, *Cronobacter muytjensii*, *Cronobacter dublinensis*, *Cronobacter condiment*, and *Cronobacter universalis* (Iversen et al. 2008; Joseph et al. 2012). Three new species (*Enterobacter pulveris*, *Enterobacter helveticus* and *Enterobacter turicensis*) that were suggested by Brady et al. (2013) to be new species based on multi-locus sequence typing (MLST) analysis were subsequently found to belong to two new genera by Stephan et al. (2014) based on new genome-scale analyses. Among the 7 *Cronobacter* species, *C. sakazakii* showed a much higher isolation frequency than the other species, accounting for 72.1 % of all *Cronobacter* isolates in the *Cronobacter* PubMLST database (http://www.pubmlst.org/cronobacter/) (Almajed and Forsythe 2016).

Members of the genus *Cronobacter* exhibit greater stress tolerance than other common pathogens, such as *Escherichia coli* and *Salmonella*, especially to osmotic and desiccation conditions (Breeuwer et al. 2003; Burgess et al. 2016). It was reported that *C. sakazakii* can survive on dehydrated powdered infant formula (PIF) for over than 2.5 years, which is the longest survival time out of the *Cronobacter* species and other common pathogens (Barron and Forsythe 2007). Therefore, *C. sakazakii* is extensively present in the environment and a frequent contaminator of food. The ability of *C. sakazakii* to survive in PIF is a substantial threat to neonates and infants because they are often highly dependent on PIF. Surviving *C. sakazakii* is capable of rapidly multiplying to dangerous levels during reconstitution before feeding, and it can cause serious clinical symptoms, include necrotizing enterocolitis (NEC), bacteremia and meningitis, resulting in fatality rates ranging from 40 to 80 % (Bowen and Braden 2006; Friedemann 2009). At least 27 deaths are known to have occurred out of approximately 120 cases that have been documented around the world up to July 2008 (FAO/WHO 2008).

Adhering to epithelial cells is a crucial step that *C. sakazakii* must complete to cause pathogenic disease. To increase our understanding of the pathogenesis of this bacterium, several studies have attempted to explore the adhesive and invasive capabilities of the pathogen in different human-derived cell lines in vitro. Mange et al. (2006) first assessed the adhesive properties of different *C. sakazakii* strains to two epithelial cell lines and a brain microvascular endothelial cell line. They found that the adhesive capabilities varied among the strains and that adhesion was non-fimbrial-based. Using a rat cell line, Townsend et al. (2007) were the first to compare the invasive capabilities of different *C. sakazakii* strains in capillary endothelial cells. In 2008, they studied adhesion and invasion in epithelial cells and endothelial cells in isolates obtained during a French outbreak in 1994 (Townsend et al. 2008). A study by Giri et al. revealed that certain *Cronobacter* isolates can invade and translocate across cultured human intestinal epithelial cells and brain microvascular endothelial cells (Giri et al. 2012). Recently, Almajed and Forsythe (2016) experimentally demonstrated that *C. sakazakii* clinical isolates possess a strong capacity to invade and translocate through human colonic epithelial cells (Caco-2) and brain microvascular endothelial cells. All of these studies macroscopically demonstrate the adhesive and invasive capabilities of *C. sakazakii* strains. Nevertheless, we currently know very little about how these processes occur at a molecular level. Outer membrane protein A (OmpA) is a well-characterized factor that plays an important role in adhesion to and invasion of endothelial cells (Nair et al. 2009). Another factor, outer membrane protein X (OmpX), was also shown by Kim et al. (2010) to be crucial to invasion into human enterocyte-like epithelial and intestinal epithelial cells. Although some progress has been made in furthering our understanding of the adhesive and invasive properties of *C. sakazakii*, the mechanisms that underlie these pathogenic processes largely remain unknown.

In the current study, to further our understanding of the molecular mechanisms involved in adhesion to and invasion into host cells in *C. sakazakii*, we constructed a Tn5 transposon mutant library and screened it to identify mutants that showed defects in adhesion or invasion. We used these mutants to identify functional genes that are potentially responsible for adhesion or invasion. We then performed Raman spectroscopy to analyze variation in the biochemical components of the mutants. Our data shed light on a variety of molecular characteristics of *C. sakazakii* at the biochemical component level. Finally, we performed real-time PCR to further explore the relative expression levels of genes shown to contribute to adhesion or invasion.

## Materials and methods

### Bacteria and culture conditions

A total of 56 *C. sakazakii* strains were used in the current study, including 4 strains that were purchased from American Type Culture Collection (ATCC) and 52 strains that were isolated from food samples that were produced in 13 countries (Table 1). All of the food-origin strains were collected by different Entry–Exit Inspection and Quarantine Bureaus of China from 2005 to 2010. The isolates were classified as *Cronobacte*r species using the API 20E, VITEK or Biolog system and further identified as *C. sakazakii* using 16S rDNA sequencing and 10 phenotypic tests.

**Table 1:** The *C. sakazakii* strains used in this study

No.	Strain	Origin/source
1	ATCC12868	ATCC/USA
2	ATCC29004	ATCC/USA
3	ATCC29544	ATCCUSA
4	BAA894	ATCC/USA
5	ENS60309-1	Skimmed milk powder/India
6	ENS60607	Milk powder/Ireland
7	ENS70101	Skimmed whey powder/USA
8	ENS70115	Whey powder/France
9	ENS70208	Whey powder/India
10	ENS70216	Powdered infant formula/China
11	ENS70307-2	Milk powder/China
12	ENS70510	Milk powder/USA
13	ENS70817	Milk powder/China
14	ENS70819	Whey powder/USA
15	ENS70819-2	Whey powder/USA
16	ENS71106	Milk powder/New Zealand
17	ENS71123	Skimmed milk powder/Canada
18	SAKA080704-1	Whey powder/Netherlands
19	SAKA080704-2	Whey powder/Netherlands
20	SAKA080721	Skimmed milk powder/USA
21	SAKA081013-2	Milk powder/Australia
22	SAKA081021	Whey powder/New Zealand
23	SAKA081104	Milk powder/Australia
24	SAKA081111	Whey powder/USA
25	SAKA090109	Whey powder/USA
26	SAKA090225	Cake flour/USA
27	SAKA090303	Whey powder/New Zealand
28	SAKA090309	Whey powder/Netherlands
29	SAKA090310-1	Milk powder/France
30	SAKA090318	Cream cheese/Australia
31	SAKA090505	Milk powder/New Zealand
32	SAKA090814	Milk powder/Australia
33	SAKA100322	Milk powder/New Zealand
34	SAKA100531	Wheat cereal/USA
35	SAKA100607	Milk powder/Netherlands
36	SAKA10119	Milk powder/New Zealand
37	SAKA10120	Milk powder/New Zealand
38	SAKA10128-91	Pig’s head/Spain
39	SAKA10208	Milk powder/Singapore
40	SAKA10315	Milk powder/New Zealand
41	SAKA10506-1	Whey powder/France
42	SAKA10506-2	Whey powder/France
43	SAKA110519-164	Unknown/Laboratory
44	SAKA110609-1	Unknown/Laboratory
45	SAKA110609-2	Unknown/Laboratory
46	SAKA110609-3	Unknown/Laboratory
47	SAKA80220A	Powdered infant formula/Netherlands
48	SAKA80221	Whey powder/Austria
49	SAKA80222	Whey powder/France
50	SAKA80408	Milk powder/Australia
51	SAKA80417-1	Casein Protein Powder/Ireland
52	SAKA90807	Milk powder/New Zealand
53	SAKA90930	Milk powder/China
54	SAKA91019	Milk powder/Australia
55	SAKA91021	Milk powder/New Zealand
56	SAKA91218	Milk powder/New Zealand

All of the strains were cultured in Luria-Bertani (LB) broth at 37 °C overnight while shaking (180 rpm) and then transferred into fresh LB broth at ratio of 1:100 for further culturing until the OD_600_ value reached 0.6–0.8.

### Epithelial cell cultures

HCT-8 cells (ATCC CCL-244; American Type Culture Collection, Manassas, Virginia) were grown in 24-well tissue culture plates containing RPMI-1640 medium (Invitrogen, Carlsbad, CA, USA) supplemented with 10 % heat-inactivated fetal bovine serum (Gibco, Carlsbad, CA, USA), 50 IU/mL penicillin, and 50 μg/mL streptomycin. The plates were incubated at 37 °C in 5 % CO_2_ until approximately 90 % of each well was covered by the cells.

### Adherence or invasion assays

The adherence and invasion abilities of *C. sakazakii* against HCT-8 cells were analyzed according to methods described in previous reports (Mange et al. 2006; Quintero et al. 2011), with some modifications. HCT-8 monolayer cells were incubated in 24-well plates and then washed with prewarmed phosphate-buffered saline (PBS) three times. Different *C. sakazakii* strains were collected in log phase using centrifugation at 3000×*g* for 5 min and then washed once with PBS buffer. The bacterial cells were resuspended in RPMI-1640 medium to achieve an OD_600_ value of 0.1. A 0.5 mL volume of bacterial suspension was added to each well of the plates, and the bacteria were allowed to interact with the epithelial cells for 3 h at 37 °C in 5 % CO_2_. After the incubation period, the plates were gently washed with prewarmed PBS 3 times to remove non-adhered bacterial cells. Adhered or invaded *C. sakazakii* cells were released from the plates by adding 0.5 mL/well 0.1 % Tritonx-100 and counted using the plating method described by the manufacturer for chromogenic cronobacter isolation agar (Oxoid, Basingstoke, UK). Empty wells that did not contain HCT-8 cells were used as the controls to calculate the number of bacterial cells that specifically adhered to the epithelial cells. Adherence assays were performed in triplicate (n = 3) using each *C. sakazakii* strain, and each assay was tested in duplicate (n = 2).

### Mutant generation and adhesion and invasion analyses

The isolate SAKA10119 showed the best ability to adhere to HCT-8 cells, and it was therefore selected to generate transposon mutants using an EZ-Tn5™ Transposome Kit (Epicenter, Madison, USA). Briefly, a single colony of the SAKA10119 strain was inoculated into LB broth and cultured overnight at 37 °C while shaking. The overnight cultures were diluted 1:100 into 30 mL fresh LB broth and incubated until OD_600_ reached 0.4-0.6. Lysozyme was added to the bacterial cells at a final concentration of 10 μg/mL and the cells were then incubated at 37 °C for 30 min to improve transformation efficiency. The treated cells were cooled on ice for 30 min and then collected via centrifugation at 1500×*g* for 10 min at 4 °C. The cell pellets were washed with ice-cold sterile water and glycerol (10 %, V/V), and then resuspended in 1 mL of glycerol (10 %, V/V). To construct a mutant library, the Tn5 transposon was electrically transformed into SAKA10119 cells according to the manufacturer’s instruction. The mutants colonies that appeared on the LB plates containing 50 μg/mL kanamycin were subsequently cultured in LB broth and used in HCT-8 cell interaction assays according to the method described above. Each mutant was analyzed in duplicate in each assay, and each experiment was performed in triplicate.

### Identification of transposon insertion sites

In the mutants that showed a significant decrease in the ability to adhere to HCT-8 cells, the Tail PCR method was used to identify the transposon insertion sites (Du et al. 2012). Briefly, DNA was extracted from each mutant using QIAamp DNAmini Kit (Qiagen, Hilden, Germany) according to the manufacturer’s instructions, and the purified DNA was then used as a template for a two rounds of nested PCR amplification. The obtained fragments were cloned into the pMD18-T vector (Takara, Dalian, China) and prepared for DNA sequencing using vector-specific primers. The DNA sequences that flanked the Tn5 transposon were identified using Blast analysis of the data available in the NCBI (The National Center for Biotechnology Information) database.

### Raman spectroscopy analysis

Raman spectroscopy analyses were performed according to methods described in a previous report (Wang et al. 2015). Briefly, monoclonal colonies of the parent strain and the 8 mutant strains were inoculated into 5 mL of LB broth and cultured at 37 °C overnight while shaking (180 rpm). The cultures were transferred into 5 mL of fresh LB broth and culture under the same conditions until the OD_600_ value reached 0.6–0.8. One milliliter of each logarithmic phase bacterial culture was collected via centrifugation at 6000×*g* for 5 min. The cell pellets were rinsed 3 times using PBS (pH = 7.4), and the cells were finally suspended in 1 mL of sterile deionized water. Five microliters of each bacterial suspension were transferred to a gold-coated glass slide (Thermo Scientific Inc., Waltham, MA, USA), and the slide was allowed to dry at room temperature. The samples were analyzed using a Renishaw inVia Raman system (Renishaw, Gloucestershire, UK) equipped with a 785-nm near-infrared diode laser and a Leica microscope (Leica Biosystems, Wetzlar, Germany). Raman scattered light was collected using a CCD array detector (576- by 384-pixel) during exposure to an incident laser (300 mW). Raman spectra were collected using a 50× objective over a 10-s exposure time with a wave number shift range of 400–1800 cm^−1^. Ten spectra were collected for each strain, and experiments were performed in triplicate. Matlab (The MathWorks, Inc., Natick, MA, USA) was used to construct chemometric models based on the wavenumbers that were obtained between 400 and 1800 cm^−1^. Unsupervised principal component analysis (PCA) was performed to investigate chemical variations in the bacterial samples by constructing a cluster-based segregation model (Huang et al. 2004).

### Real-time PCR analysis

The 8 putative adherence- or invasion-related genes that were screened in the current study were submitted to real-time PCR analysis to determine their mRNA expression levels both before and after the bacteria were allowed to adhere to epithelial cells. HCT-8 cells were cultured in 75-cm^2^ tissue flasks until about 90 % of the bottom of the flask was covered by monolayer cells. The SAKA10119 strain was cultured to logarithmic phase and washed 3 times with PBS buffer. The bacterial cells were then resuspended in RPMI-1640 medium, and 15 mL of the suspension was added to the epithelial cells. After the cells were incubated at 37 °C for 3 h in 5 % CO_2_, the adhered bacteria and the epithelial cells were lysed using 0.1 % (w/v) sodium dodecyl sulfate, 0.1 % (V/V) acidic phenol, and 19 % (V/V) ethanol in water for 30 min on ice (Lucchini et al. 2005). Total RNA was extracted from the mixture using a Bacterial RNA Kit (Omega Bio-Tek, Norcross, GA, USA). The total RNA that was extracted from the SAKA10119 strain that did not interact with epithelial cells was used as the control. cDNA was reverse-transcribed using an ImProm-II™ Reverse Transcription System (Promega, Madison, WI, USA). Eight pairs of primers specific for the 8 genes and a pair of primers specific for the 16S rDNA gene, which was used as an internal reference, were synthesized (Table 2) and used to perform qRT-PCR in a RealPlex 4 Master Cycler (Eppendorf, Hamburg, Germany).

**Table 2:** Primers used for real-time PCR

Gene	Primer name	Primer sequence	Product size (bp)
*recB*	AFK64_02315 F	CAGGATACCGACCCGCAACA	116
	AFK64_02315 R	TTTATGCGTTTCGCGGTGC	
*bglF*	AFK64_20760 F	CGGCATCAGCATGTCATAG	139
	AFK64_20760 R	CTTTAGTGTCTCGGCGAGTTT	
*fnr*	AFK64_08415 F	AATCCCGTTCGAGACGTTAG	104
	AFK64_08415 R	TCATATCCTGGTCGCCTTTG	
*flhA*	AFK64_06715 F	CGCATCCGCAGTATCCGTAA	129
	AFK64_06715 R	GCCGATTTCCACGCCTTTC	
*fliR*	ESA_02516 F	CAATCTTGACGCGGATGCTAA	102
	ESA_02516 R	ACTGGCTGACTCTGTACTTCTGG	
*hp*-*1* ^a^	AFK64_01330 F	GTTTCATATTCTTCGAGCTTTGG	119
	AFK64_01330 R	TTGCCCGACAATCTTGTGCC	
*hp*-*2* ^a^	ESA_04202 F	CTCAGCCGGGTGTTTTCACT	106
	ESA_04202 R	GCGCTAATTCTGCCAGCAA	
*hp*-*3* ^a^	ESA_00132 F	CGTTTTACGGGCTTGTCTGT	104
	ESA_00132 R	ACCGCCTGGCAATAAATCA	
*16S rDNA*	16S F	ACCCGCAGAAGAAGCAC	148
	16S R	GCAGTTCCCAGGTTGAG	

^a^ *hp*-*1*, *hp*-*2* and *hp*-*3* indicate the three hypothetical proteins coding genes

The strain SAKA10119 has been deposited in China Center for Industrial Culture Collection (CICC) under accession number CICC24112. The 16S rRNA gene sequence of the strain SAKA10119 has been deposited in GenBank database under accession number KX237756.

## Results

### Adherence or invasion analyses of different strains

**Fig. 1:**
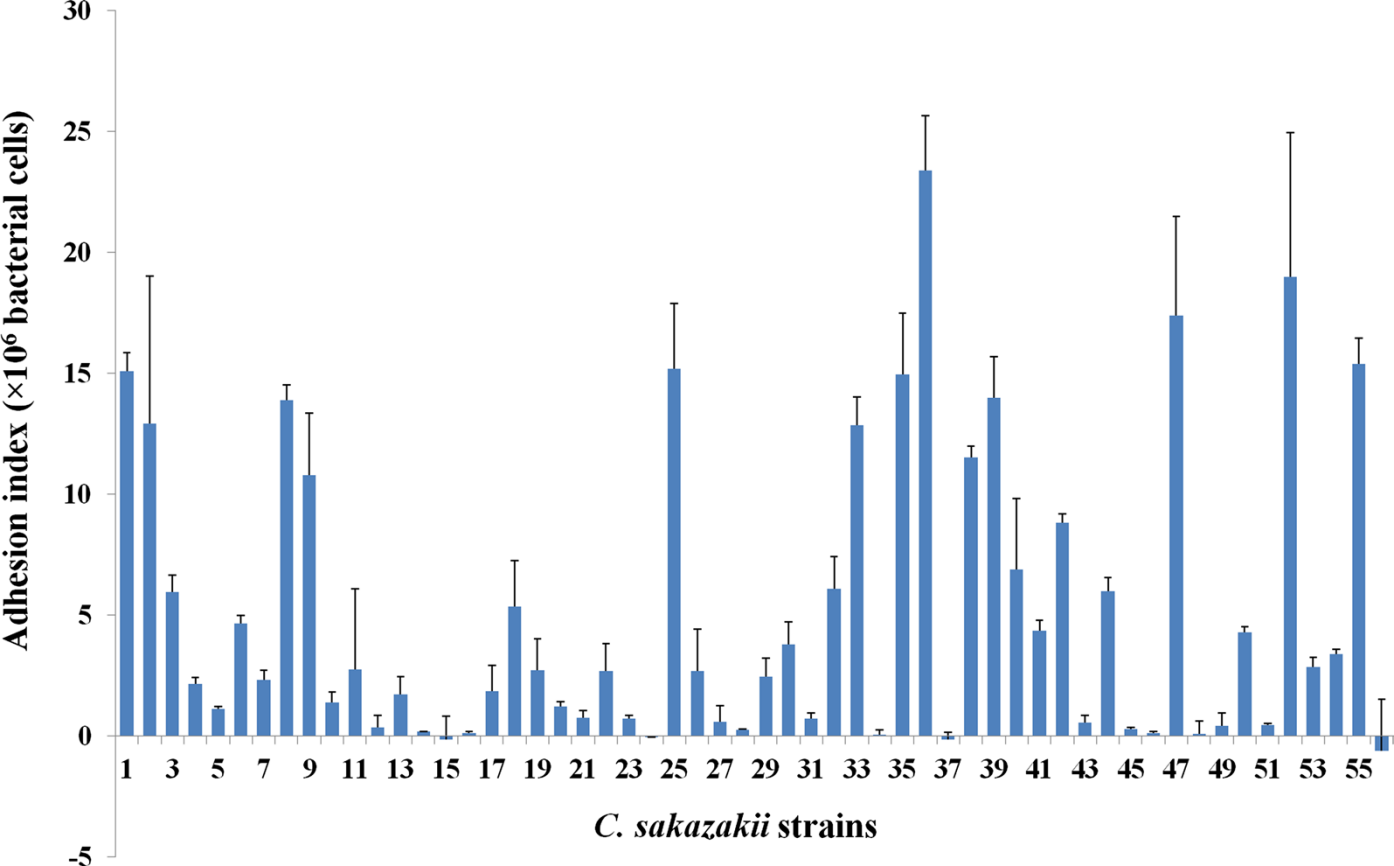
Comparison of adhesion or invasion by different *C. sakazakii* isolates in human epithelial cells. The *numbers* 1–56 indicate the numbers of the strains as they are listed in Table 1. The ability of each strain to adhere or invade is represented as the difference between the number of bacteria that adhered to or invaded into epithelial cells and number of bacteria that adhered to the empty tissue culture plate

A total 56 strains, including food isolates and standard strains, were analyzed to determine their ability to adhere to and invade intestine epithelial cells. The adhesive or invasive abilities of each strain were described using an interaction index that indicates the difference between the number of bacteria that adhered to or invaded the epithelial cells and the number of bacteria that adhered to the empty wells of the tissue culture plate. As is shown in Fig. 1, 13 strains exhibited stronger adherence capacities than the rest of the strains. The interaction indexes of the 13 strains were all above 1.0 × 10^7^ CFU. Among these strains, the strain SAKA10119 showed the strongest capacity to adhere to epithelial cells, with an adhesion index that reached 2.3 × 10^7^ CFU. A total of 24 strains showed adhesion indexes between 1 × 10^6^ and 1 × 10^7^ CFU. The adhesion indexes of the other 19 strains were lower than 1 × 10^6^ CFU, demonstrating their comparatively weaker ability to adhere or invade the cells. Among these strains, 4 showed a similar or even weaker ability to adhere to epithelial cells than to the empty plate.

### Construction and analysis of the mutants

**Fig. 2:**
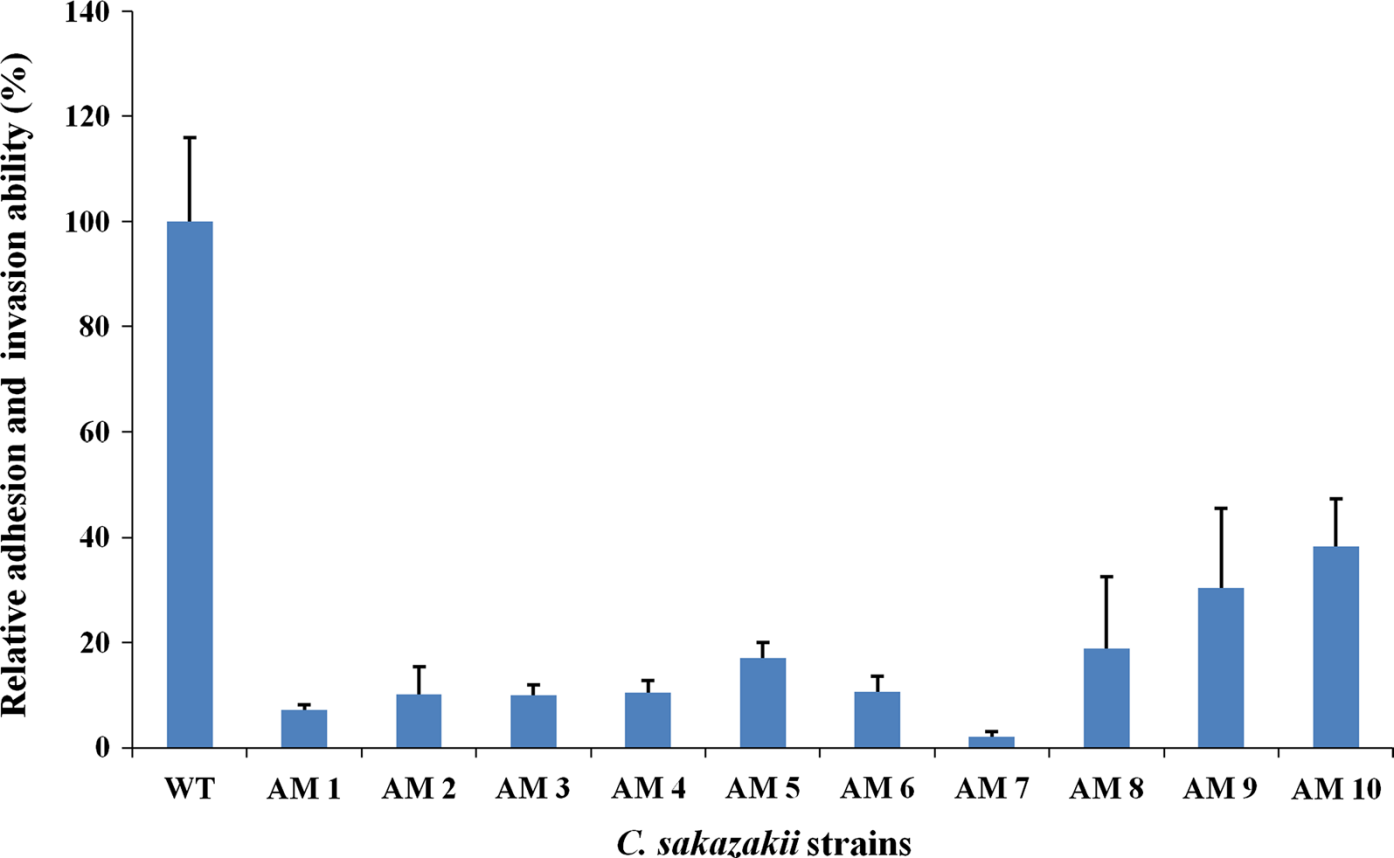
Adhesion or invasion by the wild type and mutant strains when incubated with epithelial cells. WT is the wild type strain. AM 1–10 are the 10 mutants that displayed defective adhesion or invasion against epithelial cells. The reductions in adhesion or invasion that were observed in each of the mutant strains are represented as percentages. These values were calculated by comparing the number of adhered or invaded bacterial cells in the mutants to the number in the wild type strain

Based on the results of our analyses of adhesion and invasion, the strain SAKA10119 was selected to construct a mutant library using the EZ-Tn5™ < KAN-2 > Transposon. A total of 1084 mutants were separated and cultured to further analyze their adhesive and invasive capabilities. PCR amplifications were performed using primers specific for the kanamycin resistance gene on the Tn5 transposon to evaluate the quality of the mutant library, and the results showed that a specific band was amplified from all 24 of the randomly selected colonies (Additional file 1: Figure S1). These data indicate that the mutation library was successfully constructed. The capacity of the 1084 mutant cell lines to either adhere to or invade epithelial cells was analyzed, and the results were compared to the results for the parent strain. Most of the mutants showed an adherent ability that was similar to that of the wild type strain. However, 10 of the mutants (designated AM 1–10, meaning Adhesion or invasion defective Mutants) presented a higher than 50 % reduction in adherence in comparison to the adherence observed in the parent strain (Fig. 2). These 10 mutants were therefore selected to identify the interrupted genes.

### Identification of Tn5 transposon locations

The flanking regions of the Tn5 transposon were targeted using Tail-PCR. After two rounds of amplification, fragments ranging from approximately 500–1500 bp were obtained from the 10 adhesion- or invasion-defective mutants. These 10 fragments were sequenced, and eight different genes showing a high similarity to sequences in the *C. sakazakii* genome were obtained (the same 190 bp sequence was obtained from 3 mutants). These genes encoded an exonuclease subunit, a phosphotransferase system (PTS) sugar transporter, a transcriptional regulator, two flagellar biosynthesis proteins, and three hypothetical proteins (Table 3). Seven out of the eight genes were located on the chromosome, and 1 was located on a plasmid (AM 4, which had a mutation in a PTS sugar transporter encoding gene). Seven of the genes were disrupted in the coding region, and one was disrupted in the upstream non-coding region (AM 10, which was mutated in the upstream region of a hypothetical protein encoding gene).

**Table 3:** Mutants with reduced adhesion or invasion capabilities and the affected genes

Mutants	Length obtained (bp)	Gene and locus tag	Source	Putative function	Identities (%)
AM 1-3	184	*recB,* AFK64_02315	*C. sakazakii,* NCTC 8155	Exonuclease V subunit beta	177/184 (96)
AM 4	510	*bglF,* AFK64_20760	*C. sakazakii,* NCTC 8155	PTS sugar transporter (beta-glucoside component)	493/510 (97)
AM 5	340	*fnr,* AFK64_08415	*C. sakazakii,* NCTC 8155	Fumarate and nitrate reduction regulatory protein	337/340 (99)
AM 6	323	*flhA*, AFK64_06715	*C. sakazakii,* NCTC 8155	Flagellar biosynthesis protein FlhA	320/323 (99)
AM 7	315	*fliR,* ESA_02516	*C. sakazakii,* ES15	Flagellar biosynthesis protein FliR	313/315 (99)
AM 8	94	*hp*-*1* ^a^, AFK64_01330	*C. sakazakii,* NCTC 8155	Hypothetical protein	91/94 (97)
AM 9	190	*hp*-*2* ^a^, ESA_04202	*C. sakazakii,* ATCC BAA-894	Hypothetical protein	189/190 (99)
AM 10	772	*hp*-*3* ^a^, ESA_00132	*C. sakazakii,* ATCC BAA-894	Hypothetical protein	759/772 (98)

^a^ *hp*-*1*, *hp*-*2* and *hp*-*3* indicate the three hypothetical proteins coding genes

The mutants AM 1-3 had the same insertion in the coding region of the gene *recB*, which encodes a subunit of exonuclease V. These three mutants showed 89.8–92.8 % less adhesion or invasion than the parent strain (Fig. 2). In AM 4 and AM 5, the Tn5 transposon had inserted into the coding regions of a PTS sugar transporter gene and a transcriptional regulator gene, respectively. The PTS sugar transporter gene was the only gene that was located on a plasmid. Disruptions in two of the genes resulted in an 89.5 and 82.9 % reduction in adhesion or invasion (Fig. 2). The mutants AM 6 and AM 7 had disruptions in different flagellar biosynthesis genes, both of which contained the Tn5 transposon in their coding regions. These two mutants showed 89.3 and 97.9 % less adhesion or invasion than the wild type strain (Fig. 2). The mutant AM 7 contained a disrupted *fliR* gene and exhibited the weakest adherence to epithelial cells among all of the mutants. The remaining three mutants (AM 8–10) were disrupted in genes encoding hypothetical proteins. The mutant AM 10 was different from the others because the insertion took place in the upstream coding region of the gene. These three mutants (AM 8, 9 and 10) displayed 18.8, 30.4 and 38.3 %, respectively, of the adhesion or invasion capacity that was observed in the wild type strain (Fig. 2).

### Raman spectroscopy analysis

**Fig. 3:**
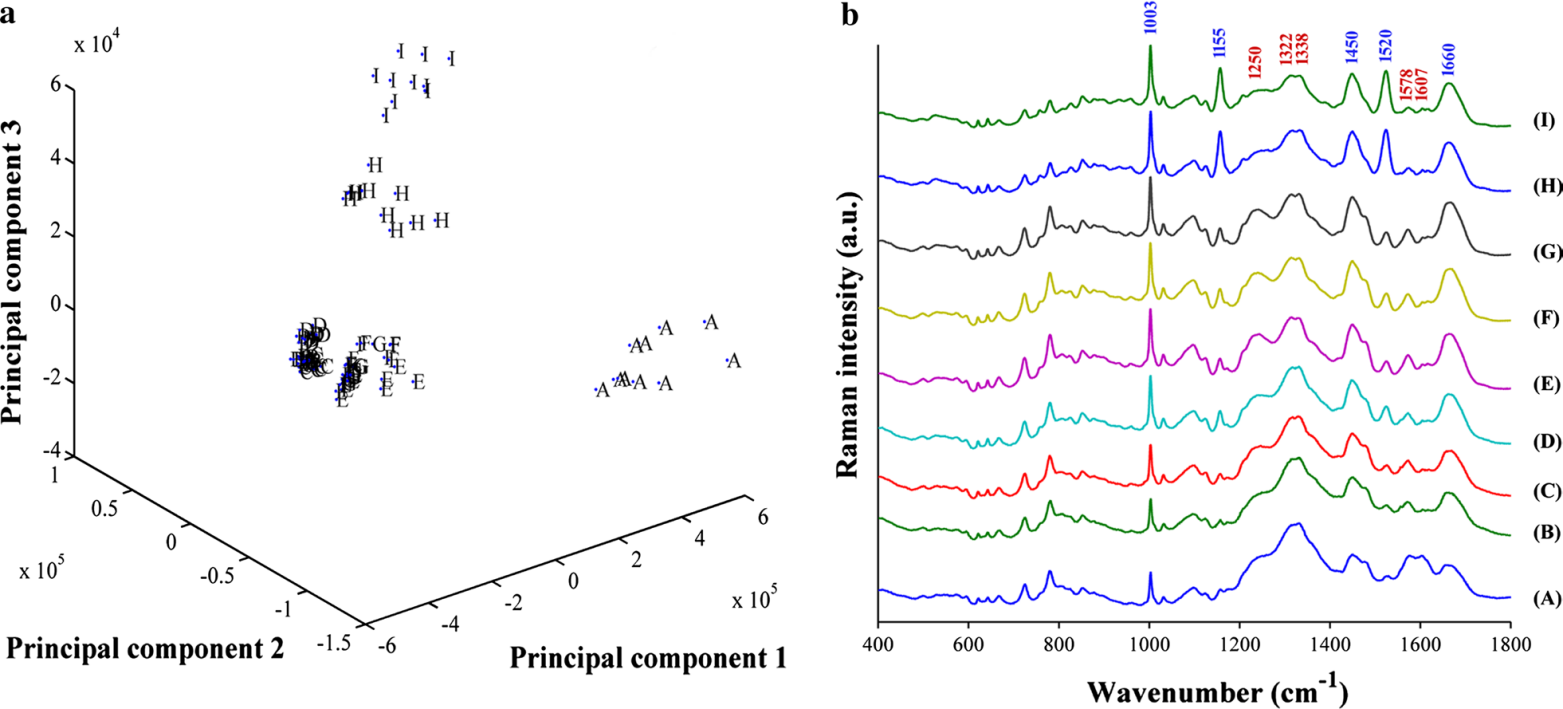
Raman spectroscopy analysis of the *C. sakazakii* wild type strain and each of the mutants. **a** A principal component analysis (PCA) model that was based on the Raman spectral features of the wild type strain and the mutants. **b** A comparison of the Raman spectral features between the wild type and mutant strains. A: wild type strains; B–I: mutants AM 2, AM 6, AM 4, AM 5, AM 7, AM 8, AM 9 and AM 10, respectively. The *numbers* in *red* indicate the decreases observed in biochemical components, and the numbers in *blue* indicate the increases observed in biochemical components

Raman spectroscopy was used to analyze variations in the biochemical components of the eight *C. sakazakii* mutants (Only AM 2 was selected from the three mutants with an identical insertion site) in comparison to the wild type strain. A PCA model was established to differentiate the wild type and mutant strains, and the results indicated that there was a clear segregation between the wild type strain and the mutants (Fig. 3a). The AM 9 strain, the AM 10 strain, and the rest of the mutants formed three distinct groups, each of which demonstrated a different set of variations in their biochemical components.

A comparison of the Raman spectra showed that five peaks were lower (1250, 1322, 1338, 1578 and 1607 cm^−1^) and five peaks were higher (1003, 1155, 1450, 1520 and 1660 cm^−1^) in the mutants than in the wild type strain (Fig. 3b). The assignments of the peaks were determined according to methods described in previous studies (De Gelder et al. 2007; Xie et al. 2005). The results indicated that the defects in the adhesive capacity of the mutants were related to decreases in protein amide III (1250 cm^−1^), protein -CH deformations (1322 and 1338 cm^−1^), nucleic acids (1578 cm^−1^) and tyrosine (1607 cm^−1^) and increases in phenylalanine (1003 cm^−1^), carotenes (1155 and 1520 cm^−1^), and fatty acids (1450 and 1660 cm^−1^). The mutants AM 9 and AM 10 exhibited the most significant differences from the wild type strain, demonstrating that the two genes (ESA_04202 and ESA_00132) that were interrupted in these strains are strongly associated with the biosynthesis of the above components. The mutants AM 4, AM 5, AM 7 and AM 8 showed comparatively smaller changes in these biochemical components when compared to the results for mutants AM 9 and AM 10. The remaining two mutants (AM 2 and AM 6) showed the weakest variations in comparison to the wild type strain. The decrease in the biosynthesis of tyrosine that was observed in these two mutants was similar to that observed in other mutants.

### Real-time PCR analysis

**Fig. 4:**
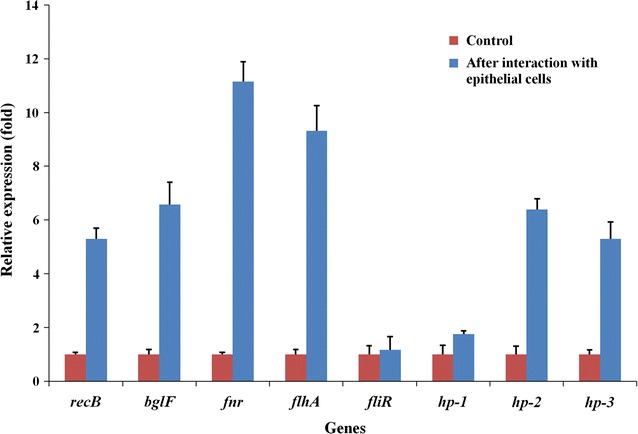
Real-time PCR analysis expression of the eight genes before and after interaction with epithelial cells. The change in the expression of each gene is represented as the ratio of the expression level of the gene after the bacteria interacted with epithelial cells to the level in the bacteria without interaction with epithelial cells. *hp*-*1*, *hp*-*2* and *hp*-*3* indicate the three hypothetical proteins coding genes

Real-time PCR was performed to analyze variations in the mRNA expression levels of the genes that were identified in the current study. We examined their expression before and after the bacteria were allowed to adhere to epithelial cells. The results indicated that the transcription of all eight of the genes was upregulated 1.2 ± 0.5- to 11.2 ± 0.7-fold (Fig. 4), demonstrating the relevance of these genes to the adhesiveness or invasiveness of *C. sakazakii*. Among these 8 genes, the transcriptional regulator gene *fnR* was upregulated the most, and the flagellar biosynthesis protein gene *fliR* was upregulated the least.

## Discussion

The adhesion and invasion of *C. sakazakii* in epithelial cells is the first critical step in establishing a successful systemic infection (Amalaradjou et al. 2014). Although many studies have been performed to analyze the adhesiveness and invasiveness of *C. sakazakii* in epithelial cells in vitro, the molecular mechanisms underlying these processes are poorly understood. Our recent report about variations in the transcriptome of *C. sakazakii* after adhesion to or invasion into human epithelial cells shed light on these molecular mechanism (Jing et al. 2016). However, too many candidate genes were identified in that study, and much more experimental evidence was needed to better verify the roles of the screened genes. The Tn5 transposon is an efficient tool that can be used to identify the genes that are responsible for specific phenotypic variations, and it has been widely used to screen genetic systems that are involved in biofilm formation (Hartmann et al. 2010; Du et al. 2012), responses to osmotic stress (Alvarez-Ordóñez et al. 2014a) and acid stress (Alvarez-Ordóñez et al. 2014b), tolerance to serum (Schwizer et al. 2013), and yellow pigmentation (Johler et al. 2010) in *C. sakazakii*. In the current study, the Tn5 transposon was used to construct a mutant library to screen for genes that might potentially be responsible for the adhesion and invasion capabilities of *C. sakazakii* in epithelial cells.

The ability to adhere and invade are widely thought to be critical pathogenic properties of *C. sakazakii*, but little data is currently available on this subject, except for studies on a limited number of factors, such as OmpA and OmpX. In the current study, a total of 8 genes were experimentally shown to be responsible for adhesion or invasion in *C. sakazakii*. These included an exonuclease V subunit beta gene (*recB*), a PTS sugar transporter gene (*bglF*), a transcriptional regulator gene (*fnr*), two flagellar biosynthesis genes (*flhA* and *fliR*) and 3 hypothetical protein encoding genes.

*recB* encodes a beta subunit of the bacterial RecBCD enzyme, which possesses DNA helicase and nuclease activities and is involved in a major pathway during homologous recombination that particularly contributes to repairing double stranded (ds) DNA breaks induced by damage (Wu et al. 2012). In the trimeric RecBCD enzyme, the RecB subunit functions with 3′ to 5′ helicase and translocase activities (Taylor et al. 2014). This gene therefore appears to be unrelated to bacterial adhesion or invasion capacities. However, a *recB*-like gene that was identified in *Helicobacter pylori* was shown to play a role in DNA repair and host colonization (Wang et al. 2009). Mutations in the gene resulted in the fragmentation of genomic DNA and cell death within a short period of time and reduced colonization in host stomachs. Their work seemed to show that the gene has putative functions in adhesion or invasion when the bacterium invades host cells. However, it seems more reasonable to attribute the reduction in colonization to one of the phenotypes that was caused by an impaired ability to repair DNA. It is reasonable that the putative correlation between the *recB* gene and the adhesion or invasion capabilities of *C. sakazakii* might be explained in the same way.

PTS is carbohydrate uptake system that is responsible for selectively transporting sugar molecules across the inner bacterial membrane while simultaneously catalyzing sugar phosphorylation (McCoy et al. 2015). Many studies have shown that this gene family is involved in virulence between bacterial pathogens and their hosts. Rouquet et al. (2009) identified an operon that encoded three subunits of PTS transporters in *E. coli* and showed that it was involved in adherence to host cells and the internalization of the bacterium in different human and chicken cells. *E. coli* strains with mutations in sugar transporter genes in the PTS showed attenuated colonization in the guts of mice and attenuated virulence in mouse models of sepsis and pyelonephritis (Martinez-Jéhanne et al. 2012). A similar function of the gene was identified in the current study.

Fumarate nitrate reduction (FNR) regulator is important to the virulence of pathogens when they encounter variations in the availability of O_2_ (Green et al. 2014). FNR can sense a reduction in or the absence of O_2_ and activate functional genes that are required for anaerobic respiration in addition to virulence genes during host colonization and infection. In hypoxic niches within a host, FNR can activate the expression of cytolysin in many pathogens, such as *E. coli* and *Salmonella* (Lithgow et al. 2007; Fuentes et al. 2008). In *Shigella*, FNR mediates the type three secretion system (T3SS), which is essential for cell invasion and virulence, when the pathogen encounters variation in oxygen concentrations in the gastrointestinal tract (Marteyn et al. 2010). The *fnr* mutant of *E. coli* results in severe defects in adherence to and the invasion of bladder and kidney epithelial cells and was highly attenuated in a mouse model of urinary tract infections (Barbieri et al. 2014). Based on these studies, it is more likely that interrupting the *fnr* gene in *C. sakazakii* substantially affected the survival of the bacterium rather than affecting the cells solely by weakening their adhesive or invasive capabilities. However, further experimental evidence is required to answer this question.

It is widely recognized that flagellar biosynthesis-related proteins play major roles in the colonization of the intestinal tract or invasion into host intestinal cells by many bacterial pathogens (Barrero-Tobon and Hendrixson 2014; Stevenson et al. 2015). FlhA is a component of the export apparatus required for flagellum assembly and has been reported to play roles in adhesion to and invasion into epithelial cells in many bacteria, such as *Bacillus cereus*, *Bacillus thuringiensis* and *Pseudomonas aeruginosa* (Fleiszig et al. 2001; Ramarao and Lereclus 2006). FliR is a component of the type III flagellar export apparatus, which is mainly responsible for exporting most of the structural components of the flagellum. It has been reported to be associated with adhesion to and pathogenicity against host cells in *Leptospira interrogans* (Ruan et al. 2008). However, the functions of these two genes have not been studied in *C. sakazakii*. This is first study to explore their involvement in the adhesive or invasive properties of *C. sakazakii*.

In addition to the genes mentioned above, three genes encoding 3 small hypothetical proteins (67, 111, 113 amino acids long, respectively) were also identified in the current study. These genes have putative functions in adhesion to or the invasion of epithelial cells in *C. sakazakii*. The identification of the unknown functions of these genes would be helpful for determining the basis of the pathogenicity of this opportunistic pathogen.

Raman spectroscopy is a powerful technique that is used to characterize and image biochemical components in living cells (Schie and Huser 2013). In the current study, Raman spectroscopy analysis of mutants with defective adhesiveness or invasiveness against epithelial cells showed that they contained less protein amide III, protein -CH deformations, nucleic acids and tyrosines. These results indicate that the mutants exhibited depressed protein and nucleic acid biosynthesis, reflecting the inhibited life activities of the mutants. Conversely, the levels of phenylalanine, carotenes and fatty acids were found to be negatively associated with adhesion and invasion in *C. sakazakii*. These findings do not clarify the mechanisms that underlie the adhesiveness and invasiveness of *C. sakazakii* against host cells, but these results do shed light on the mechanisms that are involved at a biochemical component level in the bacterium.

In summary, in the current study, eight genes were identified and found to be associated with the adhesive or invasive characteristics of *C. sakazakii* in human epithelial cells. Raman spectroscopy was used to investigate variations in the biochemical components in the mutants that were caused by the interruption of the eight genes. Real-time PCR was performed to further confirm the relatedness of these genes to interactions between *C. sakazakii* and host cells. The data presented in this study will be useful in determining the mechanisms underlying the pathogenesis of *C. sakazakii*.

## Additional file

10.1186/s13568-016-0246-4 Quality evaluation of the mutant library using PCR specific for the kanamycin resistance gene in the Tn5 transposon. M, 100 bp DNA ladder; 1-24, kanamycin resistant gene fragments that were amplified from randomly selected clones in the mutant library.

## Abbreviations

MLST: multi-locus sequence typing
PIF: powdered infant formula
NEC: necrotizing enterocolitis
OmpA: outer membrane protein A
OmpX: outer membrane protein X
ATCC: American Type Culture Collection
LB: Luria–Bertani
PBS: phosphate-buffered saline
PCA: principal component analysis
CICC: China Center for Industrial Culture Collection
AM: adhesion or invasion defective mutants
PTS: phosphotransferase system
FNR: fumarate nitrate reduction
