# Supplementary material for: Screening of genes involved in interactions with intestinal epithelial cells in Cronobacter sakazakii
Source: AMB Express. 2016 Sep 15;6:74. doi: 10.1186/s13568-016-0246-4 (PMC5023641; doi:10.1186/s13568-016-0246-4)
Supplement: Supplementary file 1 — 10.1186/s13568-016-0246-4 Quality evaluation of the mutant library using PCR specific for the kanamycin resistance gene in the Tn5 transposon. M, 100 bp DNA ladder; 1-24, kanamycin resistant gene fragments that were amplified from randomly selected clones in the mutant library. [file 13568_2016_246_MOESM1_ESM.pdf]

Journal: AMB Express

Screening of genes involved in interactions with intestinal epithelial cells in *Cronobacter sakazakii*

Xin-jun Du[1,a], Xia Zhang[1,2,a], Ping Li[1], Rui Xue[1], Shuo Wang[1]*

[1]Key Laboratory of Food Nutrition and Safety, Ministry of Education, Tianjin University of Science and Technology, Tianjin 300457, China

[2]Tianjin Entry-Exit Inspection and Quarantine Bureau, Tianjin 300461, China

*Corresponding author: Key Laboratory of Food Nutrition and Safety, Tianjin University of Science and Technology, Tianjin 300457, China. Tel.: +86 22 60912484; fax: +86 22 60912484.

E-mail address: s.wang@tust.edu.cn (S. Wang)

[a]These authors contributed equally to this work.

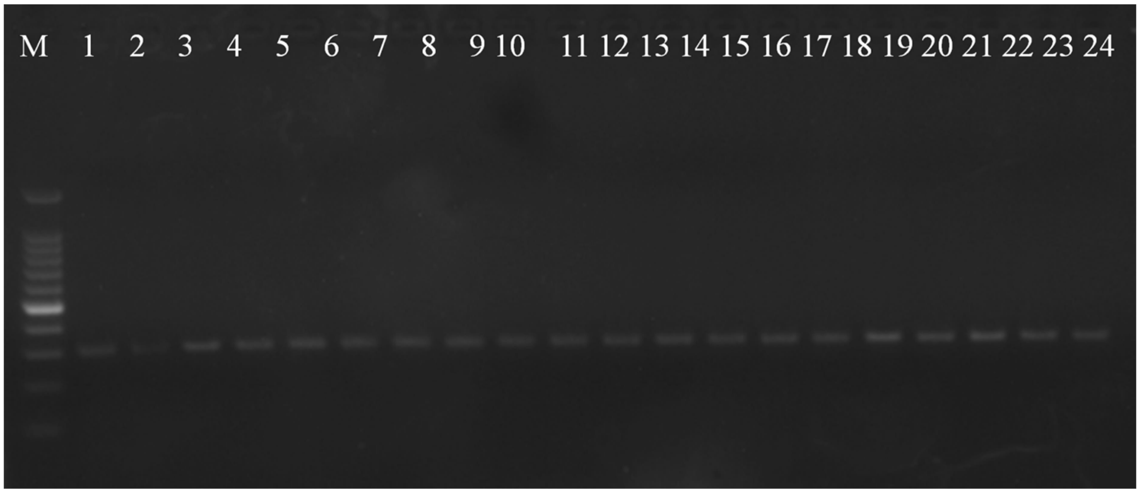

Fig. S1 Quality evaluation of the mutant library using PCR specific for the kanamycin resistance gene in the Tn5 transposon. M, 100 bp DNA ladder; 1-24, kanamycin resistant gene fragments that were amplified from randomly selected clones in the mutant library.
